# The Caribbean, Central and South America Network for HIV Epidemiology: 20 Years of HIV Research in the Region

**DOI:** 10.1002/jia2.70169

**Published:** 2026-07-25

**Authors:** Jessica L. Castilho, Stephany N. Duda, Bryan E. Shepherd, Timothy R. Sterling, Anna K. Person, Peter F. Rebeiro, William C. Wester, John R. Koethe, Jesse Carlson, Rachael A. Pellegrino, Karu Jayathilake, Fernanda Maruri, Hilary Vansell Riley, Amanda Garcia De Matos Amaral, Marina Cruvinel Figueiredo, Paridhi Ranadive, Megan Turner, Gustavo Amorim, Vickie Myers, Heather Burgess, Shengxin Tu, Liping Du, Danni Shi, Zhouhui Liang, Kaixing Liu, Chao Yan, Beatriz Grinsztejn, Valdilea G. Veloso, Paula M. Luz, Sandra Wagner Cardoso, Ruth Friedman, Ronaldo I. Moreira, Monica Derrico Pedrosa, Hugo Perazzo, Rodrigo Moreira, Maria Pia Diniz Ribeiro, Mario Sergio Pereira, Emilia Moreira Jalil, Thiago Silva Torres, Carolina Coutinho, Mayara Secco Torres Silva, Flaviana Pavan Victoriano, Jorge Pinto, Flavia Ferreira, Marcelle Maia, Victoria Bocardi, Julia Caporali, Flavia Fonseca, Mateus Westin, Regina Célia de Menezes Succi, Daisy Maria Machado, Aida de Fátima Barbosa Gouvêa, Fabiana Bononi do Carmo, Claudia P. Cortes, Maria Fernanda Rodriguez, Gabriel Castillo‐Rozas, Fabio Paredes, Georgina Estadella, Jean William Pape, Vanessa Rouzier, Adias Marcelin, Jodany Bernadin, Stanley Cadet, Marco Tulio Luque, Diana Varela, Magda Chavez, Ada Mailhot, Eduardo Gotuzzo, Fernando Mejia, Gabriela Carriquiry, Claudia Nuñez Mochizaki, Brenda Crabtree Ramirez, Juan Sierra Madero, Yanink Caro Vega, Álvaro López Iñiguez, Ana Fernanda Ramos Menchelli, Paola Alarcón Murra, Geovanna Coello, Guadalupe Muñuzuri Nájera, Rodrigo Ville Benavides, Jessica Mejía, Távata Bejarano, Nancy Sierra, Valeria Fink, Florencia Cahn, Ines Aristegui, Maria Ines Figueroa, Nicolas Doudtchitzky, Javier Mariani, Mariela Ceschel, Gissella Mernies, Agustin Nava, Carina Cesar, Pedro Cahn, Caribbean, Central and South America network for HIV Epidemiology (CCASAnet) of the International epidemiology Databases to Evaluate AIDS (IeDEA)

**Affiliations:** ^1^ Vanderbilt University Medical Center Nashville Tennessee USA; ^2^ Instituto Nacional de Infectologia Evandro Chagas Fiocruz Rio de Janeiro Brazil; ^3^ Universidade Federal de Minas Gerais Belo Horizonte Brazil; ^4^ Universidade Federal de São Paulo São Paulo Brazil; ^5^ Fundación Arriarán Santiago Chile; ^6^ Universidad De Chile Santiago Chile; ^7^ Les Centres GHESKIO Port‐au‐Prince Haiti; ^8^ Hospital Escuela Universitario Tegucigalpa Honduras; ^9^ Instituto Hondureño de Seguridad Social Tegucigalpa Honduras; ^10^ Instituto De Medicina Tropical Alexander von Humboldt, Universidad Peruana Cayetano Heredia Lima Peru; ^11^ Instituto Nacional de Ciencias Médicas y Nutrición Salvador Zubirán Mexico City Mexico; ^12^ Fundación Huésped Buenos Aires Argentina

**Keywords:** Caribbean region, cohort studies, HIV, Latin America, observational studies

## Abstract

**Introduction:**

Latin America is a key region in advancing efforts to end the HIV epidemic globally. Despite breakthroughs in HIV treatment and prevention in the last 20 years, as well as region‐wide improvements in health infrastructure and policies, there has been a 13% increase in new acquisitions in Latin America from 2010 to 2024. The Caribbean, Central and South America network for HIV epidemiology (CCASAnet), established in 2006, is one of seven regions in the International epidemiology Databases to Evaluate AIDS (IeDEA). CCASAnet contributes substantially to regional science and the development of national and region‐wide policies. Here, we describe data sources and operational methodologies employed by CCASAnet, as well as the current state of the cohort.

**Methods:**

CCASAnet data are collected using standardized data elements, including demographic information, HIV disease history, laboratory data, antiretroviral regimens, co‐infections and comorbidities. Data collection has expanded to include prospective data such as substance use, antiretroviral therapy adherence, geriatric syndromes and tuberculosis treatment. All clinical sites retain ownership of their data, and CCASAnet data contribute as able to global IeDEA‐level projects. Robust data quality initiatives and statistical methods have strengthened the cohort.

**Results:**

CCASAnet consists of nine clinics across seven countries (Argentina, Brazil, Chile, Haiti, Honduras, Mexico and Peru). Over 61,000 adults and children living with HIV have contributed observational data through December 2023. While CD4 at enrolment has remained largely unchanged, time to antiretroviral therapy initiation has decreased, retention in care has improved and the proportion with undetectable HIV RNA has dramatically increased. Co‐infections and AIDS‐defining malignancies remain a cause of morbidity and mortality, but non‐communicable diseases have also increased. Overall mortality trends in the region have improved over time, with some notable exceptions.

**Conclusions:**

Underscoring the importance of maintaining longitudinal collaborations and data collection, CCASAnet remains the largest source of high‐quality data for HIV epidemiology in Latin America and serves as an important evidence base for informing global and regional policy and stakeholder decisions related to the HIV epidemic.

## 1 Introduction

Ending the HIV epidemic in Latin America is an ongoing challenge; despite region‐wide improvements in health infrastructure and policy, there was a 13% increase in new acquisitions from 2010 to 2024 [1]. HIV incidence is increasing among men who have sex with men, transgender women, people who inject drugs, adolescents and migrant populations [2, 3]. The region represents an integral part of the global HIV picture, with 2.5 (2.2−2.8) million adults and children living with HIV in 2025 [4]. Studies have reported that although antiretroviral therapy (ART) coverage has increased, disparities and late presentation to care persist, with one‐third of people with HIV (PWH) diagnosed with a CD4 cell count (CD4) <200 cells/µL [5, 6, 7, 8]. Alongside interruptions in treatment and care, these delays continue to drive the occurrence of opportunistic infections and AIDS‐defining malignancies [9]. Tuberculosis (TB) is also a growing problem in the region, where the number of TB deaths in PWH increased by 7% between 2010 and 2017 [10]. Longer life expectancy and an ageing population of PWH are also changing the healthcare landscape, with causes of morbidity and mortality shifting from infectious to non‐communicable diseases (NCDs) such as cardiovascular disease, diabetes mellitus, hypertension and chronic liver or kidney disease [11, 12, 13, 14, 15]. Impactful and actionable regional HIV epidemiology research that explores and validates these trends remains critical.

The Caribbean, Central and South America network for HIV epidemiology (CCASAnet) was established in 2006 as one of seven geographic regions of the U.S. National Institutes of Health's International epidemiology Databases to Evaluate AIDS (IeDEA) [16] to describe the HIV epidemic in the Latin American and Caribbean region [17]. As part of the global IeDEA consortium, individual‐level retrospective and prospective CCASAnet data as well as periodic site‐level surveys about offered services are combined with data from regions around the world to investigate global trends in HIV epidemiology and care. CCASAnet has grown to include nine clinical research sites located in Argentina, Brazil, Chile, Haiti, Honduras, Mexico and Peru, along with a Data Coordinating Center at Vanderbilt University Medical Center (VUMC) in the United States.

**FIGURE 1:**
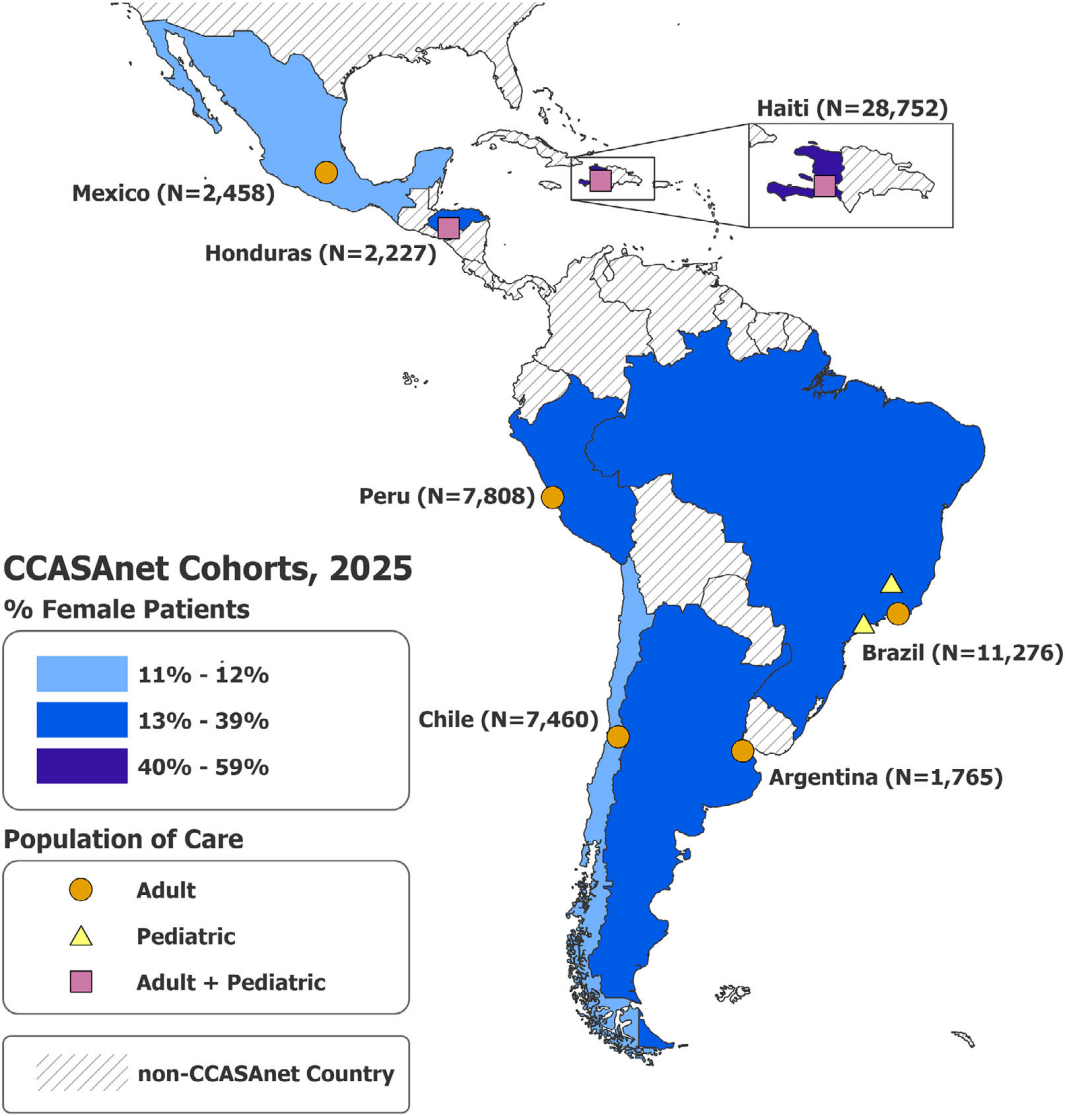
Choropleth map of the CCASAnet cohort, 2025. CCASAnet adult and paediatric care sites are indicated by differing site marker shapes and colours. Countries are shaded by percentage of female patients included in the cohort and labelled with population sizes (*N*) aggregated across all cohort sites within each country. CCASAnet, Caribbean, Central and South America network for HIV epidemiology.

CCASAnet is the largest cohort of individual‐level, quality‐controlled data available in the region, with a combined “core” database of approximately 61,000 adults and children with HIV (Figure 1). Participating sites include health clinics, public hospitals and academic medical centres, led by internationally recognized HIV clinician‐researchers. CCASAnet's role as a source of high‐quality observational HIV data is also due to a longstanding quality assurance programme [18]. On this 20th anniversary of CCASAnet, we reflect on what has been achieved over the years, present the current state of the cohort and share lessons learned from HIV cohort research in Latin America.

## 2 Methods

### 2.1 Sourcing and Sharing of Cohort Data

Following local ethics committee requirements, data for CCASAnet are extracted from routine electronic and paper health records by clinic staff at participating sites using standardized definitions and a common data model [19, 20]. Data elements include demographics, HIV history (including routes of acquisition, diagnosis dates, clinic visits, and CD4 and HIV‐1 RNA viral load [VL] values), ART history, other diagnoses such as TB or cancer and mortality. Deaths are ascertained through medical records and, where available, national and regional registry linkage. Newer data elements include pregnancy, anthropometrics and other laboratory information, when available. Sites send datasets at least annually to the Data Coordinating Center for data quality review and merging. The resulting database facilitates regional studies and global investigations with other IeDEA regions. Data access is restricted in compliance with data privacy regulations of participating countries, and data are pseudonymized prior to sharing.

For variables less reliably available in routine clinical records, CCASAnet prospectively collects data and outcomes using standardized case report forms and study protocols with informed consent determined by local ethics committees. Examples include substance use and ART adherence questionnaires being integrated into routine clinical care and frailty and cognition being assessed in a selection of older PWH. Further, CCASAnet participates in IeDEA prospective studies of adolescents and young adults with HIV, adults at risk for NCDs and PWH with TB.

All clinical sites retain ownership of their data, determination of their use and opportunities for leadership of, collaboration in and approval of all scientific products using their data. Ethics committees at each participating site and the Data Coordinating Center provide oversight and approval for data collection methods and data uses (VUMC IRB #060284).

### 2.2 CCASAnet Cohort Description

We used individual‐level demographic, diagnostic, laboratory and medication data for common indicators of HIV care from the retrospective CCASAnet dataset spanning the life of the cohort through December 2023, the latest date through which quality‐confirmed data were available. To describe temporal trends in cohort enrolment, we calculated the total number of patients with any recorded visit and the total number of new patients enrolled annually across all sites. To describe the age distribution of the cohort, we evaluated the median age annually, at enrolment and overall. To describe the health status of participating PWH, we calculated median CD4 at enrolment, median CD4 overall and proportion of the cohort with AIDS at enrolment. Finally, to describe cohort treatment outcomes, we summarized the proportion of the cohort prescribed ART, the proportion with suppressed VL (VL<500 copies/mL given changing laboratory thresholds over time) and ART regimen used among individuals initiating ART annually.

### 2.3 CCASAnet Scientific Impact

Since 2007, CCASAnet data, researchers and resources have supported nearly 300 publications ranging from site‐level investigations to global collaborative reports, from HIV clinical outcomes, epidemiologic descriptions, prospective cohorts and biostatistical methodology. As a comprehensive, systematic review of all CCASAnet studies is beyond the intent of this manuscript, we summarized recurrent themes and impactful outcomes of our 20‐year collaboration and future scientific priorities.

## 3 Results

CCASAnet sites reflect noteworthy heterogeneity and public health strengths of the region (Table 1). From seven countries, the nine clinics include five adult HIV clinics, two adult and paediatric HIV clinics (GHESKIO‐Haiti and IHSS/HE‐Honduras) and two HIV paediatric clinics (UFMG and UNIFESP in Brazil). The composition of CCASAnet sites has evolved over time, and inclusion criteria for sites into the research consortium have been described previously [21]. With the exception of GHESKIO‐Haiti, which is funded by international sponsors, sites are domestically funded public clinics that provide ART and HIV care free of charge to all residents in their location. They are a mix of community‐based outpatient clinics and hospital‐based referral centres. Currently, patients at all clinics have access to integrase strand transfer inhibitor (INSTI)‐based (specifically, dolutegravir‐based) first‐line therapies, including dual therapy. While CD4 and VL measurements are widely available, current practice at many sites reflects less frequent CD4 monitoring once clients are stable on ART. HIV pre‐exposure prophylaxis (PrEP) is offered at six sites and other medical and social services are offered at nearly all sites. Many CCASAnet sites also have electronic medical records to support patient care and leverage national HIV‐related registries to augment their clinical data. Most clinics also engage community input, including community advisory boards and peer support and education programmes.

**TABLE 1:** Programme characteristics of sites participating in CCASAnet.

	Argentina	Brazil	Brazil	Brazil	Chile	Haiti	Honduras	Mexico	Peru
Site, city	Fundación Huésped Buenos Aires	Instituto Nacional de Infectologia Evandro Chagas (INI)—Fiocruz, Rio de Janeiro	Universidade Federal de São Paulo (UNIFESP), São Paulo	Universidade Federal de Minas Gerais (UFMG), Belo Horizonte	Fundación Arriarán, Santiago	Les Centres GHESKIO Port‐au‐Prince	Instituto Hondureño de Seguridad Social and Hospital Escuela, Tegucigalpa	Instituto Nacional de Ciencias Médicas y Nutrición Salvador Zubirán, Mexico City	Instituto de Medicina Tropical Alexander von Humboldt and Universidad Peruana Cayetano Heredia, Lima
Year joined CCASAnet	2006	2011	2012	2013	2006	2006	2006	2007	2006
Clinic setting	Outpatient care	Outpatient care and hospital‐based	Outpatient care	Outpatient care	Hospital‐based	Outpatient care	Hospital‐based	Hospital‐based	Hospital‐based
Clinic cohort	Adult	Adult	Paediatric	Paediatric	Adult	Adult and paediatric	Adult and paediatric	Adult	Adult
Year of “Treat All” era start	2015	2013	2013	2013	2016	2016	2016	2015	2015
First‐line ART regimens (2025)	Adults: TDF+XTC+DTG BIC/TAF/FTC 3TC/DTG	Adults: TDF+3TC+DTG	Adults: TDF+3TC+DTG Children: first month: ZDV+3TC+RAL second month: ZDV+3TC+DTG third month to 12 years old: ABC+3TC+DTG >12 years old: TDF+3TC+DTG	Adults: TDF+3TC+DTG Children: first month: ZDV+3TC+RAL second month: ZDV+3TC+DTG third month to 12 years old: ABC+3TC+DTG >12 years old: TDF+3TC+DTG	Adults: TDF+3TC+DTG	Adults: TDF/3TC/DTG TAF/3TC/DTG Children: ABC/3TC/DTG	Adults: TDF+3TC+DTG Children: ABC+3TC+DTG	Adults: BIC/TAF/FTC DTG/3TC	Adults: TDF/3TC/DTG
Guidelines for ART initiation	National Argentine Society for Infectious Diseases	Brazilian Guideline 2024	Brazilian Guideline 2024	Brazilian Guideline 2024	National guidelines (Chile)	National guidelines (Haiti, adapted from WHO)	National guidelines (Honduras, adapted from WHO)	National guidelines (Mexico)	National guidelines (Peru)
Electronic medical record	Available	Available	Available	Available	None	Available	None	Available	None
CD4 monitoring	At baseline, every 6−12 months if viral load is undetectable, and as needed otherwise	At baseline, every 6−12 months if viral load is undetectable, and as needed otherwise	At baseline, every 6 months if viral load is undetectable, and as needed otherwise	At baseline, every 6 months if viral load is undetectable, and as needed otherwise	Baseline, every 6 months if viral load is undetectable, and as needed otherwise	Baseline and every 12 months	Baseline, Every 12 months if viral load is undetectable, and every 6 months otherwise	Baseline and every 6 months	Baseline, every 12 months if undetectable viral load, and as needed otherwise
HIV viral load monitoring	At baseline, every 6−12 months if viral load is undetectable, and as needed in viral failure	At baseline, every 6 months if viral load is undetectable, and as needed in viral failure	At baseline, every 6 months if viral load is undetectable, and as needed in viral failure	At baseline, every 6 months if viral load is undetectable, and as needed in viral failure	At baseline, every 6 months if viral load is undetectable, and as needed in viral failure	At baseline, every 12 months if viral load is undetectable, and as needed in viral failure	At baseline, every 6−12 months if viral load is undetectable, and as needed in viral failure	At baseline, every 6 months if viral load is undetectable, and as needed in viral failure	At baseline, every 12 months if viral load is undetectable, and as needed in viral failure
Viral genotyping/subtyping	After virologic failure	After virologic failure and at baseline for pregnant women and persons with TB	After virologic failure and at baseline for pregnant women and children	After virologic failure and at baseline for pregnant women, children, persons with TB, those who acquired HIV through a partner who was using ART and those who seroconvert while using pre‐exposure prophylaxis	After virologic failure	Only available occasionally through Ministry of Health support	After virologic failure	After virologic failure	Not available
HIV pre‐exposure prophylaxis services	Available	Available	Available	Available	None	Available	None	None	Available
National registries used	Health system registry	ART distribution registry, HIV lab results registry, genotyping results registry	ART distribution registry, HIV lab results registry	ART distribution registry, HIV lab results registry	HIV lab results registry, death registry	HIV registry	Not available	ART registry, death registry	Health system registry, HIV lab results registry, TB registry
Community connection	Community Advisory Board, peer support programmes	Community Advisory Board, peer educators, NGO partnerships	None	Community Advisory Board, peer educators, NGO partnerships	None	Community Advisory Board, peer educators	Yes, not specified	Community Advisory Board, peer support programmes	None

Abbreviations:3TC, lamivudine; ABC, abacavir; ART, antiretroviral therapy; BIC, bictegravir; CCASAnet, Caribbean, Central and South America network for HIV epidemiology; CD4, CD4 cell count; DTG, dolutegravir; FTC, emtricitabine; INI, Instituto Nacional de Infectologia; NGO, non‐governmental organization; TAF, tenofovir alafenamide fumarate; TB, tuberculosis; TDF, tenofovir disoproxil fumarate; UFMG, Universidade Federal de Minas Gerais; UNIFESP, Universidade Federal de São Paulo; WHO, World Health Organization; XTC, emtricitabine (FTC) or lamivudine (3TC); ZDV, zidovudine.

As of December 2023, more than 61,000 adults and children living with HIV have contributed observational data to CCASAnet (Table 2). Cohort size varies widely across sites, with nearly half of participating PWH seen at GHESKIO‐Haiti; the smallest sites are the paediatric‐only clinics. The distribution of PWH by sex reflects differences in HIV acquisition risk across the region, with more women affected in Haiti, a generalized HIV epidemic setting, compared to overwhelmingly male cohorts in Mexico and Chile, where key populations including men who have sex with men are disproportionately affected in concentrated epidemics. Patients at sites affiliated with hospitals, including sites in Mexico, Honduras and Peru, have, on average, lower CD4 nadir values than PWH at community‐based sites offering HIV screening, as in Haiti. Differences in mortality counts likely reflect both variation in overall morbidity and social vulnerability across settings as well as differences in availability of linkage to public health databases, such as those available in Brazil and Argentina.

**TABLE 2:** Total patient cohorts of interest at participating CCASAnet sites, as of 31 December 2023.

*N* (%) Median (IQR)	TOTAL	Argentina	Brazil—INI	Brazil—UNIFESP	Brazil—UFMG	Chile	Haiti	Honduras	Mexico	Peru
*N*	61,746	1765	10,270	358	648	7460	28,752	2227	2458	7808
Sex at birth										
Male	37,339 (60)	1322 (75)	7538 (73)	177 (49)	327 (50)	6589 (88)	11,925 (41)	1352 (61)	2195 (89)	5914 (76)
Female	24,407 (40)	443 (25)	2732 (27)	181 (51)	321 (50)	871 (12)	16,827 (59)	875 (39)	263 (11)	1894 (24)
Median age at enrolment	34 (26, 42)	38 (32, 46)	34 (27, 43)	3 (1, 7)	2 (1, 5)	32 (27, 40)	34 (27, 43)	32 (24, 41)	34 (28, 42)	31 (25, 40)
HIV acquisition risk factor										
MSM	16,146 (26)	395 (22)	4352 (42)	6 (1.7)	9 (1.4)	5592 (75)	228 (0.8)	344 (15)	1674 (68)	3546 (45)
Injection drug use	208 (0.3)	45 (2.5)	140 (1.4)	0 (0)	1 (0.2)	8 (<0.1)	8 (<0.1)	1 (<0.1)	5 (0.2)	0 (0)
Heterosexual	21,681 (35)	471 (27)	3934 (38)	15 (4.2)	11 (1.7)	1713 (23)	9936 (35)	1027 (46)	543 (22)	4031 (52)
Perinatal	2912 (4.7)	9 (0.5)	133 (1.3)	293 (82)	566 (87)	11 (0.1)	1439 (5.0)	295 (13)	32 (1.3)	134 (1.7)
Other	396 (0.6)	13 (0.7)	138 (1.3)	29 (8.1)	18 (2.8)	67 (0.9)	8 (<0.1)	14 (0.6)	46 (1.9)	63 (0.8)
Unknown/missing	20,403 (33)	832 (47)	1573 (15)	15 (4.2)	43 (6.6)	69 (0.9)	17,133 (60)	546 (25)	158 (6.4)	34 (0.4)
Median year of HIV diagnosis	2010 (2004, 2016)	2003 (1998, 2010)	2009 (2000, 2016)	1998 (1995, 2001)	2001 (1997, 2007)	2013 (2006, 2018)	2010 (2005, 2015)	2007 (2003, 2015)	2011 (2005, 2015)	2012 (2007, 2016)
Median year of clinic/cohort enrolment	2013 (2007, 2017)	2006 (2002, 2014)	2012 (2005, 2019)	1998 (1995, 2002)	2002 (1998, 2008)	2015 (2009, 2019)	2013 (2008, 2016)	2008 (2004, 2015)	2013 (2008, 2017)	2014 (2009, 2017)
ART naive at clinic entry	54,594 (88)	950 (54)	7586 (74)	279 (78)	606 (94)	5974 (80)	28,737 (100)	2055 (92)	1857 (76)	6550 (84)
Median CD4 at clinic entry	277 (109,484)	364 (198,552)	296 (96,539)	618 (209,1206)	800 (445, 1438)	315 (146, 496)	286 (128, 487)	175 (70, 337)	188 (55, 382)	219 (80, 391)
Median log10 HIV RNA at clinic entry	4.84 (4.11, 5.39)	4.41 (3.69, 5.04)	4.68 (3.89, 5.31)	5.14 (4.44, 5.71)	5.11 (4.31, 5.70)	4.78 (4.06, 5.34)	4.48 (3.88, 5.04)	4.70 (4.00, 5.07)	4.90 (4.45, 5.45)	5.08 (4.51, 5.54)
Median CD4 nadir	215 (79, 377)	233 (114, 379)	193 (55, 373)	184 (35, 426)	363 (168, 631)	257 (112, 417)	238 (99, 399)	132 (60, 255)	145 (41, 293)	169 (58, 323)
Deaths	8387 (14)	94 (5.3)	2315 (23)	108 (30)	95 (15)	645 (8.6)	3407 (12)	446 (20)	292 (12)	985 (13)
History of TB ever	9580 (16)	78 (4.4)	2192 (21)	83 (23)	69 (11)	302 (4.0)	4617 (16)	223 (10)	204 (8.3)	1812 (23)
Median follow‐up time, years	5.4 (1.6, 11.3)	8.2 (3.1, 16.2)	5.4 (1.0, 12.6)	9.8 (1.6, 20.8)	10.6 (3.1, 16.6)	3.8 (1.1, 8.4)	5.6 (1.8, 11.2)	6.7 (1.6, 14.2)	7.4 (2.7, 12.3)	5.2 (1.7, 9.8)

Abbreviations: ART, antiretroviral therapy; CCASAnet, Caribbean, Central and South America network for HIV epidemiology; CD4, CD4 cell count; INI, Instituto Nacional de Infectologia; IQR, interquartile range; MSM, males who have sex with males; RNA, ribonucleic acid; TB, tuberculosis; UFMG, Universidade Federal de Minas Gerais; UNIFESP, Universidade Federal de São Paulo.

**FIGURE 2:**
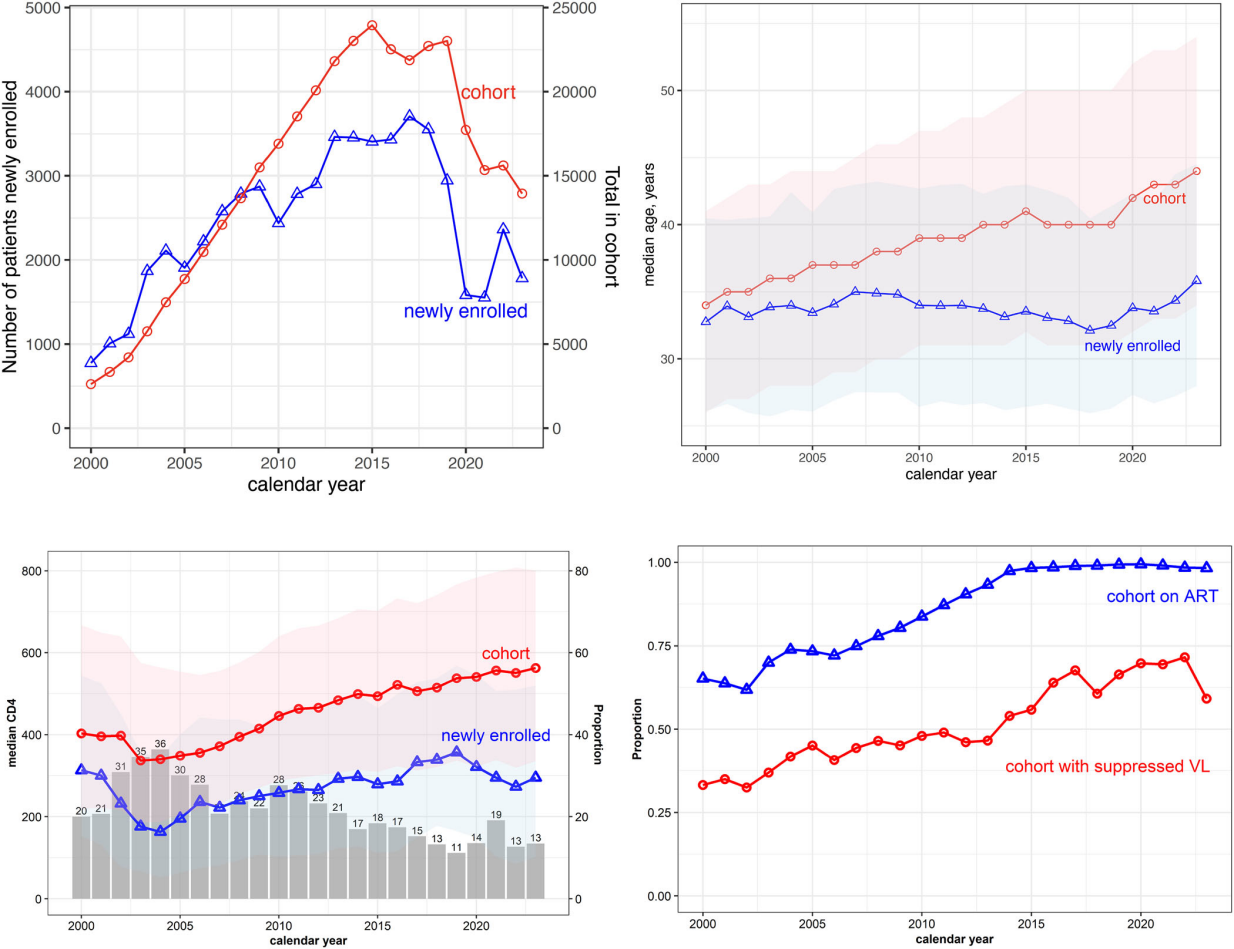
CCASAnet cohort trends, 2000−2023. The four panels show (a) CCASAnet enrolment over time, including the total number of patients with at least one recorded visit by year and total number of new patients enrolled each year; (b) median age of the cohort by year, at enrolment and overall; (c) median CD4 cell count (cells/µL) at enrolment, median CD4 overall and proportion of the cohort with AIDS at enrolment; and (d) proportion of the cohort on ART and the proportion with suppressed VL (VL<500 copies/mL at final measurement in the year) annually. ART, antiretroviral therapy; CCASAnet, Caribbean, Central and South America network for HIV epidemiology; VL, HIV viral load.

In the first two decades of 2000, the number of patients in care and new patients enrolled at sites steadily increased (Figure 2). While the median CD4 at enrolment has remained stable around 300 cells/µL, the median CD4, median age of clients enrolled in care and proportion of PWH on ART consistently increased over time, reflecting improved life expectancy due to increased ART access and use. However, rates of viral suppression have stabilized in recent years well below public health goals of 95% suppression, underscoring the need for additional interventions to address barriers to ART adherence and clinical retention. Further, since around the time of the COVID‐19 pandemic, both the number of clients newly entering care and those continuously engaged in care sharply declined, suggesting large disruptions to healthcare and social context with critical impacts on the HIV care continuum in the region.

### 3.1 Trends and Outcomes of ART in the Region

**FIGURE 3:**
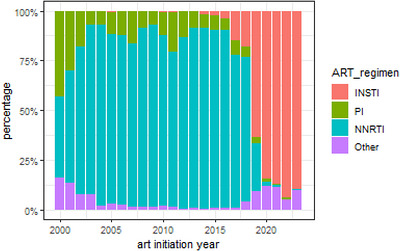
Initial ART regimen prescribed by backbone antiretroviral class among PWH at CCASAnet sites, 2000−2023. Of all PWH initiating an ART regimen at CCASAnet sites by calendar year, the proportion starting a regimen based upon a backbone regimen including an INSTI, PI, NNRTI or other medication is shown. INSTI, integrase strand transfer inhibitor; NNRTI, non‐nucleoside reverse transcriptase inhibitor; PI, protease inhibitor.

As national treatment guidelines have adopted earlier ART initiation, we have observed a trend towards more rapid ART initiation, particularly after the beginning of the “treat all” era in 2015 [6, 22]. Over time, PWH at CCASAnet sites experienced higher retention and sustained viral suppression [7], along with changing ART regimens (Figure 3). In earlier years, first‐line ART included mostly efavirenz (EFV) based ART, which demonstrated superior long‐term durability compared to protease inhibitor (PI)‐based regimens in the region [23]. While INSTI regimens now predominate at CCASAnet sites, the introduction of dolutegravir (DTG) was heterogeneous and challenged by the concurrent recommendation restricting its use in women, resulting in disparate uptake of DTG, particularly among women [24, 25].

### 3.2 HIV Continuum of Care

CCASAnet has demonstrated improvements across the HIV care continuum. In a CCASAnet study of PWH initiating ART between 2004 and 2014, 42% of PWH overall had a CD4>200 cells/µL at ART initiation, 72% were retained in care at 1 year and 77% achieved viral suppression after 1 year of ART [6]. Both ART initiation and viral suppression improved over time (*p*<0.05). A study of CCASAnet data from 2003 to 2012 showed increases in clinical retention (63%−77%), ART use (74%−91%) and viral suppression (53%−82%) [5]. Across studies, older age was consistently correlated with better retention, ART use and viral suppression, whereas injection and non‐injection drug use and alcohol use were associated with poorer retention [26, 27].

### 3.3 Infectious and Non‐Infectious Clinical Outcomes Among PWH

CCASAnet has been a pioneer in characterizing clinical outcomes of PWH in Latin America, including opportunistic infections, such as cryptococcal meningitis, TB, cancer and NCDs [15, 28, 29, 30, 31, 32, 33, 34]. TB remains a major cause of morbidity and mortality in the region, reaching a peak prevalence of 9.4% among PWH in the early 2000s [35]. NCDs (particularly dyslipidaemia, hypertension, psychiatric disorders and diabetes) and multimorbidity in people ≥50 years old in CCASAnet have increased in recent years [15]. CCASAnet also provided the first data on cancers among PWH in Latin America. Though AIDS‐defining malignancies remain the most common cancers in the region [31, 33], there has been a decline over time [31, 33, 34]. Recently, CCASAnet provided one of the first reports on cervical and anal cancer among PWH in Latin America [32].

### 3.4 Advanced HIV Disease and Mortality Trends

A CCASAnet study examining 9229 adults enrolled in HIV care between 2001 and 2014 found that 56% of PWH had advanced HIV disease (AHD, defined as CD4<200 cells/µL or clinical AIDS‐defining condition) at presentation to care [36], emphasizing the importance of early HIV diagnosis and prompt linkage to care in the region. Further, as shown in Figure 1c, AHD remains common among PWH enrolling into CCASAnet sites in recent years, and has consistently emerged as a significant predictor of mortality [36, 37, 38, 39].

Early CCASAnet studies were among the first to assess 1‐ and 5‐year all‐cause mortality in PWH in Latin America [37, 38]. Over the last two decades, mortality rates in the cohort have decreased, and life expectancies have increased [39]. Of all deaths in our cohort in the first 1, 5 and 10 years following enrolment, we found that 86%, 71% and 58% could have been averted if AHD had been prevented through early diagnosis and linkage to care [36]. Further, even among PWH achieving viral suppression, the percentage of follow‐up time individuals lived with CD4<200 cells/µL significantly increased their risk of mortality, even after adjusting for time‐updated CD4 [40]. While overall mortality trends among PWH in this region are encouraging, some countries, such as Brazil and Mexico, have reported increases in mortality in recent years, particularly from AHD [39, 41, 42]. With the continued evolution of the HIV epidemic, ongoing monitoring of mortality remains a critical tool in identifying areas for intervention.

### 3.5 Youth Living With HIV in the Caribbean and Latin America

CCASAnet was the first to examine virologic and clinical outcomes of 672 children and adolescents (<18 years of age) initiating second‐line ART regimen between 1998 and 2018, of whom 89% had perinatally acquired HIV and 48% started second‐line treatment due to previous virologic failure. Results were notable for high rates of mortality (10%) and loss to follow‐up (14%) within 3 years of second‐line ART initiation. Additionally, 32% of children went on to have another major ART regimen change in follow‐up, highlighting the clinical challenges and high morbidity and mortality in this population [43]. A prospective investigation of HIV care continuum outcomes among adolescents (ages 10−17 years) and young adults (ages 18−24 years) with HIV at CCASAnet sites observed a high prevalence of depressive symptoms (16%), recently missed ART doses (41%) and unsuppressed HIV viral load (40%), underscoring the vulnerability of youth living with HIV in the region [44]. While the incidence of vertical transmission is decreasing, ongoing HIV acquisition among adolescents and young adults as well as non‐universal usage of ART among pregnant women point to ongoing important challenges to addressing the needs of youth living with HIV in the region [45].

### 3.6 Data and Statistical Methods

Since 2006, CCASAnet has performed data audits at sites to improve data quality [46], comparing data sent to the Data Coordinating Center with clinical information in the local medical record for a random subsample of individuals [47, 48, 49]. We developed tools to streamline the audit process using the REDCap electronic data capture platform [50, 51, 52, 53], and the audit findings led to an entire area of statistical research on methods and designs for combining high‐quality (e.g. audited) data in a subsample of records with error‐prone data available in all records [54, 55, 56, 57, 58, 59, 60]. These methods are widely applicable to other settings where routinely collected data (e.g. the electronic health record) are used for biomedical research [61]. CCASAnet data have also been used to motivate and illustrate novel and impactful statistical techniques, including rank‐based regression models for handling data with detection limits (e.g. VL) [62, 63], extensions of Spearman's correlation to bivariate survival data (e.g. correlation between times from ART initiation to viral failure and major regimen change) [64, 65], techniques for covariate‐adjustment that relax linearity assumptions (e.g. natural splines) [66] and approaches for quasi‐reproducible research with sensitive data [67]. As personal health data become more difficult to share across international borders, CCASAnet has also emerged as a leader in synthetic data generation and federated learning in HIV research [68].

### 3.7 Future Research Directions

CCASAnet will continue to investigate the trends, determinants and opportunities to improve the health outcomes of PWH in the Caribbean and Latin America. Recent shifts in local care delivery, political, societal and environmental disruptions, population mobility and demographic shifts in the region each continue to impact communities living with HIV. Introduction of new HIV treatment and prevention therapies will undoubtedly affect HIV incidence, morbidity and disease outcomes. In recent years, we observed the societal, health system and individual effects of the COVID‐19 pandemic with sharp declines in patient enrolment and retention at CCASAnet clinics, decreases in average CD4 count at enrolment and recent decreases in HIV viral suppression rates (Figure 2). Ongoing and future research will seek to understand the contributors to and consequences of these trends to improve care delivery and preparedness for future crises.

## 4 Discussion

Collaborative multinational research cohorts are a powerful engine for HIV research and response. Thanks to U.S. federal funding, robust principles of collaboration and respect, commitment to science and high‐quality data, CCASAnet has been a driver of HIV research in Latin America over the past 20 years, as demonstrated through its knowledge contributions, support for national ministries of health and regional stakeholders, and training of the next generation of HIV clinician‐researchers, statisticians and data personnel. We have learned that sustained scientific partnership and high‐quality, regionally relevant research is possible, and reliable local evidence not only fills global knowledge gaps but ensures the visibility of Latin America's HIV epidemic in the global space.

CCASAnet has served as a foundation for collaboration with local and regional public health stakeholders. Sites in Brazil, Mexico and Peru have partnered with national ministries of health for the collection of registry data to augment and improve data quality. Scientific objectives and results from CCASAnet are shared with regional leaders of the Pan American Health Organization (PAHO) and, through its role in IeDEA, contribute to global reports by UNAIDS [69]. Further, the collaborations established through CCASAnet have served as the bedrock for affiliated, independent public health investigations (including a national epidemiologic study of birth outcomes associated with DTG use among women in Brazil in 2017 [25]) and novel cohort studies of critical populations and outcomes, including TB and older PWH.

Throughout two decades, CCASAnet has sustained support for early‐stage investigators. Structured training opportunities and close scientific guidance have not only enhanced local research competencies but have also contributed to the development of a new generation of regional experts. By helping to launch the careers of emerging investigators and leaders, CCASAnet has increased the visibility and understanding of the HIV epidemic in the region and informed public health responses.

Despite significant progress, CCASAnet has met persistent challenges and limitations. This manuscript highlights key lessons and recurrent themes in CCASAnet research over time but does not encompass all the research endeavours of our network over the past 20 years. Our sites provide key lessons of the HIV epidemic in their setting but are neither balanced with respect to size nor to be interpreted as representative of the HIV epidemic throughout their countries. CCASAnet sites represent important clinical research sites in their respective cities and are not meant to represent all PWH in the region given its vast diversity both within and across settings. Many are in tertiary healthcare settings and are referral centres for their regions and, thus, may include patients with greater morbidity and advanced HIV disease than PWH cared for in non‐referral clinics. Our research continues to highlight gaps in granular data for subpopulations such as young men, adolescents, women and ageing individuals, alongside inadequate surveillance of non‐AIDS‐defining comorbidities like substance‐use‐related deaths and cardiovascular diseases, which limit the epidemiologic questions that can be addressed [70]. Despite the growth in electronic health record systems in the region, many data challenges remain. Research data collection often involves paper charts due to restrictive institutional policies on secondary data use and limited systems interoperability. Similarly, valuable electronic national registry and surveillance systems in the region frequently lack access for research use and cannot be used for bulk tracing of lost‐to‐follow‐up patients or verification of mortality records, requiring manual searches, one patient at a time. Health system fragmentation, incomplete or inaccessible records and underreporting further present challenges to CCASAnet data completeness [71, 72].

Beyond structural barriers and limits in research data collection, the ongoing humanitarian crises, migration and extreme weather events disproportionately affect PWH and key populations, highlighting the need for new research approaches, including community‐led data collection, targeted interventions [73, 74, 75] and adaptive research methodologies to accurately capture evolving epidemiologic landscapes particular to the region [76]. Finally, threats to funding for international research point to the need for greater global stakeholder investment and diversification of support for HIV surveillance systems and collaborations. Although HIV care and prevention services in Latin America are overwhelmingly funded domestically [77], HIV research receives substantially less local support. Loss of international funding for HIV and infectious diseases research would require increased local investments, which may not be feasible in all settings.

Nevertheless, HIV cohort consortia such as CCASAnet continue to provide a substantial benefit in Latin America, the United States and global communities and remain essential to “closing the gaps” in HIV care continuums and outcomes.

## 5 Conclusions

In the past 20 years, CCASAnet's cohort has grown to over 61,000 adults and children with HIV. It has served as a crucial source of data for global and regional policy related to the HIV epidemic, and provided invaluable opportunities for training, mentorship and capacity‐building, while revealing important findings on the HIV continuum of care, mortality trends, opportunistic infections, NCDs and cancer. While challenges in the region remain and threats to international research collaborations and funding have created new obstacles, opportunities for collaboration in Latin America through CCASAnet and beyond must continue to advance the regional and global fight against HIV.

## Author Contributions

This paper is presented on behalf of the CCASAnet consortium. The paper was drafted collaboratively by CCASAnet investigators in a cloud‐based document editor. All sites contributed data and scientific leadership over time, as well as text for the manuscript, table content and requested data updates or clarifications for the cohort snapshot. Biostatisticians at the data coordinating centre prepared the tables and figures. The manuscript was circulated to the consortium for review. The CCASAnet multiple Principal Investigators, Jessica Castilho, Pedro Cahn and Stephany Duda, reviewed and approved the final manuscript.

## Funding

This work was supported in part by the NIH funded Caribbean, Central and South America network for HIV epidemiology (CCASAnet, U01AI069923), by grants R37AI131771, R24AI124872 and R01MH139379 from the National Institute of Allergy and Infectious Diseases, by grant R01MH139379 from the National Institute of Mental Health, by grant K99LM014428, from the National Library of Medicine and by the Tennessee Center for AIDS Research (TN‐CFAR, P30AI110527).

## Conflicts of Interest

There are no conflicts of interest to declare beyond NIH funding (money paid to institutions to support research).

## Disclaimer

The content is solely the responsibility of the authors and does not necessarily represent the official views of the National Institutes of Health. This work is subject to the NIH Public Access Policy. Through acceptance of the federal funding, the NIH has been given the right to make this manuscript publicly available in PubMed Central upon the Official Date of Publication, as defined by the NIH.

## Data Availability

CCASAnet welcomes interested investigators to collaborate with us for the use of our de‐identified, individual‐level data or (pending availability) our synthetic dataset. A data dictionary defining each field in the dataset is available upon request. In accordance with the CCASAnet Principles of Collaboration, applicants seeking data need to submit a concept sheet outlining the intended use of the data. All requests will be reviewed by the CCASAnet Executive Committee. To access the original de‐identified CCASAnet data, applicants must also complete a data use agreement with Vanderbilt University Medical Center. Applications for access to both datasets should be submitted at https://redcap.link/ccasanetsharingrequest.
